# Induction of apoptosis in human hepatocellular carcinoma cells by extracts of *Lannea coromandelica* (Houtt.) Merr. and *Diospyros castanea* (Craib) Fletcher

**DOI:** 10.1186/s13020-016-0091-z

**Published:** 2016-04-23

**Authors:** Natthida Weerapreeyakul, Cholpajsorn Junhom, Sahapat Barusrux, Thaweesak Thitimetharoch

**Affiliations:** Faculty of Pharmaceutical Sciences, Khon Kaen University, Khon Kaen, 40002 Thailand; Graduate School, Faculty of Pharmaceutical Sciences, Khon Kaen University, Khon Kaen, 40002 Thailand; Faculty of Associate Medical Sciences, Khon Kaen University, Khon Kaen, 40002 Thailand

**Keywords:** *Diospyros castanea*, *Lannea coromandelica*, Hepatocellular carcinoma, HepG2, Cytotoxicity, Apoptosis

## Abstract

**Background:**

Herbal plants are a preferred source of anticancer agents. This study aims to screen the anticancer activity of a crude extract of twigs of (a) *Bombax anceps* Pierre var. *anceps* (BA); (b) *Catunaregam tomentosa* (Blume ex DC.) Tirveng. (CT); (c) *Erythrophleum succirubrum* Gagnep. (ES); (d) *Lannea coromandelica* (Houtt.) Merr. (LC); and (e) leaves and (f) twigs of *Diospyros castanea* (Craib) Fletcher (DC).

**Methods:**

The 50 % ethanol–water extracts were prepared from each plant sample. In vitro anticancer effects of six extracts on the human hepatocellular carcinoma cell line (HepG2) in terms of cytotoxicity were investigated by neutral red assay, apoptosis induction by 4′,6-diamidino-2-phenylindole (DAPI) staining, and DNA fragmentation by agarose gel electrophoresis. Normal Vero cells were tested for comparison and to determine cancer selectivity. Gas chromatography–mass spectrometry analysis was performed to identify the compounds in the extracts.

**Results:**

The six crude extracts had different cytotoxicities and were classified into three groups based on their IC_50_ value and selectivity index (SI). DC (twig) crude extract had both a high cytotoxicity and SI toward HepG2 cells comparable to melphalan (*P* = 0.023). The crude extracts of DC (leaves), LC (twig), and BA (twig) had moderate cytotoxicity and a lower SI. Although all crude plant extracts induced apoptosis in more than 50 % of the DAPI-positive apoptotic HepG2 cells, only DC (twig) and LC (twig) showed laddering in the DNA fragmentation assay. 2-Palmitoylglycerol was the major compound common to both. Pyrogallol and lupeol were the major compounds in DC (twig) crude extract. Hexadecanoic acid and octadecenoic acid were the major compounds in LC (twig) crude extract, which had high toxicity but low selectivity.

**Conclusion:**

Ethanolic extracts from DC and LC twigs induced apoptosis in the HepG2 cell line. Pyrogallol and lupeol in DC (twig) might be responsible for the cytotoxicity toward the HepG2 cancer cells.

## Background

Hepatocellular carcinoma (HCC) in men is the fifth most common malignant tumor in the world and the second most common cause of cancer death [1]. The major risk factors for HCC include infection by hepatitis B and hepatitis C viruses, both of which are endemic in northeastern Thailand [2]. Drug resistance and severe side effects on normal tissues and cells have limited the effectiveness of chemotherapy [3].

Anticancer agents have been found in natural products, such as vincristine and vinblastine from *Catharanthus roseus* [4], taxol and docetaxel from *Taxus brevifolia* [5], and camptothecins from *Camptotheca acuminata* [6–9]. The Plant Genetics Conservation Project (under the patronage of HRH Princess Maha Chakri Sirindhorn) has reported on the cytotoxic and apoptotic effects of medicinal plants against hepatoma cells [10–13]. Subsequent study was performed on the plants locally found in Thailand which were used in this study including *Lannea coromandelica* (Houtt.) Merr. (LC); *Catunaregam tomentosa* (Blume ex DC.) Tirveng. (CT); *Erythrophleum succirubrum* Gagnep (ES); *Diospyros castanea* (Craib) Fletcher (DC); and *Bombax anceps* Pierre var. *anceps* (BA).

DC belongs to the family Ebenaceae, which is widespread in the tropics and subtropics [14]. Many Diospyros species have medicinal uses in Chinese, Ayurvedic, and African traditional medicine. Almost all the major parts of these plants have been used as medicines and reported to have biological and pharmacological activities [14]. LC belongs to the family Anacardiaceae. It is a deciduous tropical tree widely distributed in Bangladesh, India, and some other tropical countries [15–18]. CT belongs to the family Rubiaceae and has been used to treat diabetes mellitus, cancer, and tuberculosis. It possesses antimicrobial, antifungal, hypotensive, analgesic, antimalaria, antioxidant, and antileukemia pharmacological properties [19]. ES belongs to the family Leguminosae (subfamily Caesalpinioideae) and is used in Thai traditional medicines for fever and skin disease. Some isolated compounds (cassaine diterpenoid dimers) from this family contribute to anticancer activity by decreasing apoptosis inhibitors or increasing apoptosis inducers; this leads to tumor necrosis factor (TNF)-related apoptosis-inducing ligand resistance in human gastric adenocarcinoma cells [20]. BA belongs to the family Bombacaceae. Plants in this family are active against many different diseases [21–24] (Table 1); however, the dichloromethane extract from the root of *B. malabaricum* is inactive in the human epidermoid carcinoma (KB) and human cervical carcinoma (HeLa) cell lines [25].

**Table 1:** Ethnopharmacology, pharmacological activities, and taxonomic classification of plants

Scientific name (voucher specimen no.)^a^	Part of plant	Traditional uses/pharmacological activity
*Diospyros castanea* (Craib) Fletcher. (TT-OC-SK-1030) Family: Ebenaceae	Leaf	*Diospyros:* lumbago, astringent, febrifuge, anti cancer activity, anti HIV activity, anti-inflammatory activity [14]
	Fruit	*Diospyros:* carminative, astringent, cure biliousness [14]
	Seed	*Diospyros:* sedative [14]
	Bark	*Diospyros:* bitter, astringent and febrifuge [14]
*Lannea coromandelica* (Houtt.) Merr. (TT-OC-SK-993) Family: Anacardiaceae	Stem bark	LC: elephantiasis, impotence, ulcers, halitosis, heart disease, dysentery, gout rheumatism [15]
	Bark (ethanol, acetone)	LC: wound healing, antimicrobial activity in the form of simple ointments [16]
	Root bark, stem bark (crude methanol)	LC: hypotensive activity [17], *L. velutina*: antioxidation activity [18]
*Catunaregam tomentosa* (Blume ex DC.) Tirveng. (TT-OC-SK-1015) Family: Rubiaceae	Crude (ethanol)	*R. cordifolia*: diabetes mellitus, cancer, TB, antimicrobial, antifungal, hypotension analgesic, anti-malarial, antioxidant, anti-leukemic, mutagenic functions [19]
*Erythrophleum succirubrum* Gagnep. (TT-OC-SK-1082) Family: Leguminosae-Caesalpinioideae	Bark (methanol)	ES: anticancer activity [20]
*Bombax anceps* Pierre var. *anceps* (TT-OC-SK-1074) Family: Bombacaceae	Crude (methanol)	*B. ceiba*: antioxidant activity [21]
	Root (H_2_O/acetone)	*B. malabaricum*: demulcent, diuretic, restorative, aphrodisiac, emetic properties, astringent, antifungal activity, cytotoxicity, anti-HIV activity [22]
	Stem bark	*B. ceiba:* anticancer activity and anti-inflammation [23]
	Stem bark (methanol)	*B. ceiba:* antiangiogenic activity [24]

^a^TT-OC-SK Thaweesak Thitimetharoch, Orasa Chaichumporn, and Sureerat Kaewsaart

To our knowledge, the mechanism of anticancer effect of these five plants on HCC is still unknown. This study aims to screen the anticancer activity of a crude 50 % ethanol–water extract of twigs of (a) BA; (b) CT; (c) ES; (d) LC; and (e) leaves and (f) twigs of DC. The cytotoxic and apoptotic actions of the extracts were investigated with a human HCC cell line (HepG2) and compared with their effects on normal Vero cells. One advantage of using the normal Vero cell line is that Vero cell line multiplies indefinitely while primary liver cells have a limited lifetime and die after a given number of generations. Consequently, the Vero cell line is easy to handle and represents an unlimited self-replicating source with a relatively high degree of homogeneity. We then analyzed major compounds common to the different effective extracts using gas chromatography–mass spectrometry (GC–MS).

## Methods

### Chemicals and reagents

Dulbecco’s modified Eagle’s medium (DMEM), fetal bovine serum, penicillin, and streptomycin were purchased from GIBCO™ (Invitrogen, Grand Island, New York, USA). Acetonitrile (HPLC grade; Fisher Scientific, Leicestershire, UK), orthophosphoric acid (analytical grade; BHD, Merseyside, UK), and ultrapure water from a Milli-Q system (Millipore, Billerica, Massachusetts, USA) were used for the mobile phase preparation. Dimethylsulfoxide (DMSO), ethidium bromide, the fluorescence dye 4′,6-diamidino-2-phenylindole (DAPI), sodium bicarbonate (NaHCO_3_), neutral red, and a standard anticancer drug (melphalan) were purchased from Sigma-Aldrich (Darmstadt, Germany). A FlexiGene DNA kit was purchased from Qiagen (Hilden, Germany), agarose (molecular grade) was purchased from Bio-Rad (Hercules, California, USA), and a DNA ladder with stain was purchased from SibEnzyme (Novosibirsk, Russia). Isopropyl alcohol (biotechnology grade) was purchased from BioBasic Inc. (Amherst, New York, USA) and 100 bp + 1.5 Kb DNA ladder with stain was purchased from SibEnzyme (Novosibirsk, Russia). All other reagents used in this study were purchased from Sigma Chemicals Co. (St. Louis, Missouri, USA). An Annexin V-FITC Apoptosis Detection Kit was purchased from Bender MedSystems GmbH (Vienna, Austria).

### Preparation of plant extracts

Leaves and twigs of LC, CT, ES, BA, and DC were collected in Khon Kaen Province, Thailand. They were authenticated using taxonomic methods by Assistant Professor Thaweesak Thitimetharoch., Faculty of Pharmaceutical Sciences, Khon Kaen University. The descriptions of plant species were determined using the Flora of Thailand and the Thai Forest Bulletin [26–30]. The specimen vouchers were deposited at the medicinal herbarium of the Faculty of Pharmaceutical Sciences, Khon Kaen University, Khon Kaen province, Thailand. All plants were extracted and prepared using the following method. The crude 50 % ethanol–water extract was obtained as previously described [12]. Briefly, dried plants were cut and macerated with 50 % ethanol and water in a ratio of 1 g dried plant to 6 ml of extract solvent. This mixture was left for 7 days with occasional shaking. The solvent was filtered with Whatman filter paper No. 1, distilled in vacuo with a rotary evaporator below 40 °C, and freeze-dried to obtain the crude extracts. The percentage yields of the extracts and the plant parts from which they were obtained are shown in Table 2. Stock solution of each extract was freshly prepared in DMSO to make 100 mg/mL stock solution and further diluted with culture media to create a working solution. In all experiments, the concentrations of extracts (working solution) ranged from 10 to 500 μg/mL and the final concentration of DMSO was <1 % to retain the percentage cytotoxicity of DMSO at <10 %.

**Table 2:** Cytotoxicity of plant extracts in HepG2 and vero cells (mean ± SD) (n = 5)

Plant	Part used	% yield	IC_50_ ± SD (μg/mL)		SI
			HepG2	Vero	
*L. coromandelica* (LC)	Twig	6.01	307.12 ± 15.97^a^	489.37 ± 37.43	1.6^a^
*C. tomentosa* (CT)	Twig	6.88	316.70 ± 34.82	294.59 ± 76.25	0.9
*E. succirubrum* (ES)	Twig	8.94	207.89 ± 40.89	94.18 ± 29.10	0.4
*D. castanea* (DC)	Leaves	7.23	147.12 ± 10.34^a^	237.11 ± 43.03	1.6^a^
*D. castanea* (DC)	Twig	8.74	112.35 ± 18.26^b^	>500	>4.5^b, c^
*B. anceps* (BA)	Twig	5.96	212.74 ± 27.01^a^	>500	2.3^a^
Melphalan			39.79 ± 7.62	178.98 ± 15.25	4.5

^a^Moderate cytotoxicity with lower selectivity index (SI < 3.0)

^b^High cytotoxicity with higher selectivity index (SI > 3.0) to HepG2 cell lines

^c^Non-significant difference vs. melphalan (*P* > 0.05)

### Cell culture

The HepG2 cells (human HCC cell line) and Vero cells (normal kidney epithelial cells) were grown in DMEM supplemented with 10 % fetal bovine serum, 100 U/mL penicillin, and 100 μg/mL streptomycin. The cell lines were cultured in an incubator (Procell; Jencons-PLS, USA) at 37 °C in a humidified atmosphere containing 5 % carbon dioxide (CO_2_).

### Cytotoxicity assay

Cell viability was measured using a neutral red (NR) assay. Cells were seeded at a density of 3 × 10^5^ cells per well in 96-well plates (Costar 3599; Corning Inc., USA) in the medium, and incubated for 24 h. The stock solutions of plant extracts in DMSO were diluted with DMEM to the desired concentrations (10–500 μg/mL). The maximum concentration of the 50 % ethanol–water extracts in each test was 500 μg/mL, such that the final concentration of DMSO did not exceed 1 % (v/v) and the cytotoxicity of DMSO was <10 %. The control wells contained only the complete medium and cells. The cells were treated with plant extracts for 24 h before the NR solution (50 μg/mL) in media was added to each well. Cells were further incubated for 1 h at 37 °C in the 5 % CO_2_ incubator before being washed with buffer. The treated viable cells with NR were washed with 1X PBS buffer and were lysed with 100 μL of 0.33 % hydrochloric acid/isopropanol. The absorbance of NR that represented the viable cell was measured using a microplate spectrophotometer system (Anthos 2010; Biochrom, UK) with an absorbance of 520 nm and a reference wavelength of 650 nm [31]. The percentage cytotoxicity was calculated as previously described [32]. The extent of cytotoxicity toward HepG2 and Vero cells was presented as the average of half the maximal inhibitory concentration (IC_50_) of the crude extract. Melphalan was used as the positive control. The selectivity of the sample against the HepG2 cancer cells was compared with the normal Vero cells and is expressed by the selectivity index (SI). The SI is the IC_50_ ratio in Vero cells versus HepG2 cells. Any sample with an SI value greater than three is considered to have high selectivity [32].

### Determination of apoptotic induction

#### Detection of nuclear morphological changes using DAPI fluorescent dye staining assay

A DAPI staining assay was performed to identify the nuclear morphological changes in apoptotic cells as previously described [33]. Briefly, the cells were seeded at a density of 1 × 10^6^ cells per well in 24-well plates in the medium and incubated for 24 h. The cells were then treated at 2 × IC50 with each plant extract or melphalan (at the highest concentration of 500 μg/mL, 0.1 % DMSO) for 24 h. The controls were the untreated wells, which contained only the complete medium and cells. After treatment, the cells were washed with 1× PBS once and harvested using centrifugation (Daihan Scientific, Seoul, Korea) at 400×*g*, at 4 °C for 5 min. The treated cells were fixed at −20 °C for 10 min with 50 μL of methanol and water (1:1). The 100 μL (1 μg/mL) of DAPI dye was added to the frozen cells and the mixture kept at 37 °C for 30 min for staining protected from light. Excess DAPI was then removed with the supernatant by centrifugation at 400×*g* at 4 °C for 5 min. A 20 μL PBS to glycerine ratio of 1:1 was added to the cells. The cells were analyzed by dropping aliquots of them onto slides and closed with the cover slip. The fluorescent dsDNA-specific dye stain showed a fragmented nuclei pattern of apoptotic cells compared with the intact nuclei of normal cells under 40× magnification using fluorescent microscope visualization (Eclipse 80i, Nikon, Kanagawa, Japan) [33–35]. The amount of apoptotic cells was counted and the percentage calculated.

#### DNA fragmentation detection using DNA laddering assay

A DNA ladder assay was performed to identify DNA fragmentation among apoptotic cells as previously described [12]. Briefly, 1 × 10^6^ cells/well were seeded into a 24-well, flat-bottomed plate and incubated in a 5 % CO_2_ incubator at 37 °C for 24 h. After treatment of cancer cells with 2 × IC_50_ of the 50 % ethanol–water extracts or melphalan for 24 h, the cells were collected and washed with medium. The DNA in the cell pellets was extracted using a FlexiGene DNA kit. Aliquots (2 μg) of the DNA were analyzed using electrophoresis at 100 V for 40 min in 1.8 % agarose gels containing 0.1 % ethidium bromide. After electrophoresis, the DNA fragments were visualized using a UV transilluminator (Vilber Lourmat Deutschland GmbH, Eberhardzell, Germany) and gel pictures were taken with a UV-illuminated camera (Syngene, UK).

#### GC–MS analysis

GC–MS analyses were performed on selected crude ethanolic extracts to identify the chemical components. A gas chromatography system (Model 6890 N; Agilent Technologies, Shanghai, China), coupled with a mass selective detector (Model 5973 N; Agilent Technologies, Delaware, USA) and a GC auto-sampler (Agilent 7683 Automatic Liquid Sampler, Santa Clara, California, USA), was employed for all analyses. Briefly, 2 μL of crude extracts (10.40 mg of DC, 10.74 mg of LC) were injected into the GC column equipped with a capillary column (122–5532 DB-5 ms, length 30.0 m, diameter 250 μm, film thickness 0.25 µm). The injection temperature was set to 250 °C. Helium was used as the carrier gas at a constant flow rate of 2 ml/min. The oven temperature program was 80 °C for 6 min, followed by a 5 °C min^−1^ oven temperature ramp to 280 °C within 70 min. The mass spectra were recorded with a 50–500 MHz scanning range using mass spectrometry. The chromatograms and mass spectra of constituents of the crude extracts were evaluated by comparing their mass spectra with those in the database (Wiley 7N.l database, Agilent Technology, New York, USA).

### Statistical analysis

The experiments were performed in three to five replicates and data are presented as mean ± SD. The statistical significance of differences in multiple-group comparisons was evaluated with the analysis of variance (ANOVA) followed by Tukey’s honestly significant difference test using SPSS version 11.5 (SPSS Inc., Chicago, IL, USA). A two-tailed Student’s t test was also performed. *P* values less than 0.05 were considered statistically significant.

## Results

### Cytotoxic effects and SI of plant extracts on human HCC cell line

All 50 % ethanol–water crude extracts of the five plants showed a moderate to high cytotoxic effect on a human HCC cell line (HepG2). The cytotoxicity and SI values of the plant extracts for HepG2 cells are summarized in Table 2. The IC_50_ and SI of crude extract were demonstrated for the leaves of DC, twigs of DC, and LC. All of the crude 50 % ethanol–water extracts showed significantly lower cytotoxicity than melphalan (IC_50_ = 39.79 ± 7.62 μg/mL; SI = 4.5) (LC*: P* < 0.001, CT*: P* < 0.001, ES*: P* < 0.001, DC leaves*: P* = 0.002, DC twig*: P* = 0.023, and BA*: P* < 0.001, respectively). Only the crude extract of DC (twig) showed high cytotoxicity against the HepG2 cells; an effect that was significantly different to that of melphalan (*P* = 0.023).

### Determination of apoptosis pattern using DAPI

**Fig. 1:**
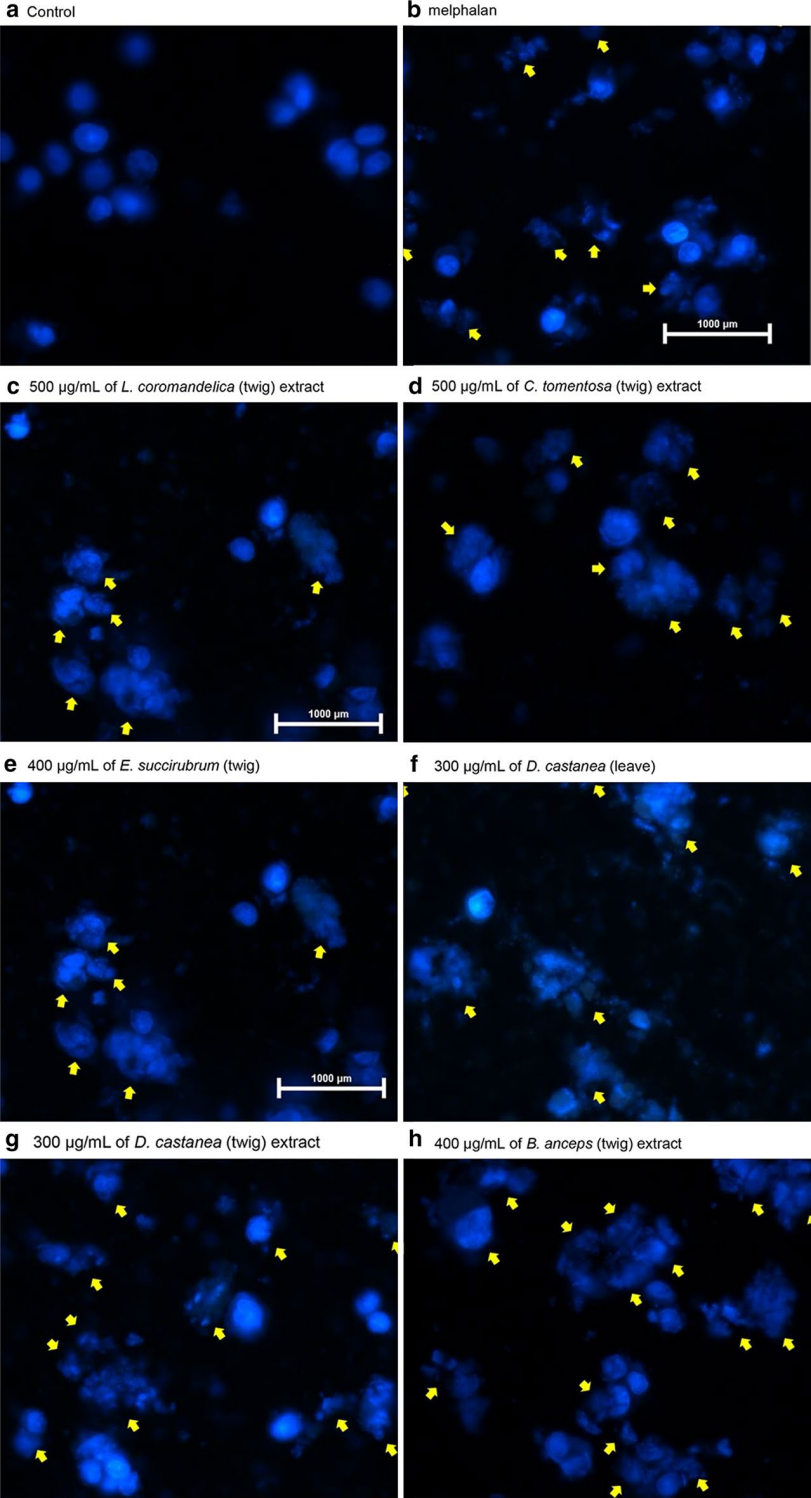
Nuclei morphological change of HepG2 cells stained with DAPI fluorescent dye at 24 h. **a** Untreated HepG2 cells; **b** 80 μg/mL of melphalan; **c** 500 μg/mL of *L. coromandelica* (twig) extract; **d** 500 μg/mL of *C. tomentosa* (twig) extract; **e** 400 μg/mL of *E. succirubrum* (twig) extract; **f** 300 μg/mL of *D. castanea* (leaf) extract; **g** 300 μg/mL of *D. castanea* (twig) extract; and **h** 400 μg/mL of *B. anceps* (twig) extract. *Arrows* indicate apoptotic nuclei based on heterogeneous staining, DNA condensation, and apoptotic bodies at ×40 objective magnification

Cells were stained with DAPI fluorescent dye to assess apoptosis 24 h after treatment of cell lines with plant extracts at 2 × IC_50_. Using 40× magnification, all five plant extracts were observed to cause alterations of nuclear morphology, revealing cell shrinkage, membrane blebbing, and apoptotic bodies (Fig. 1). The percentage of apoptotic cells was determined by observation using inverted microscopy for twigs of LC, CT, ES, BA, and leaves of DC. The positive control melphalan showed the highest induction of apoptosis in HepG2 (82.5 ± 8.0 %) and twigs of DC showed the lowest (66.0 ± 11.5 %) (Table 3).

**Table 3:** Apoptosis induction of ethanolic plant extracts in HepG2 (mean ± SD) (n = 3)

Sample	Part	Apoptosis induction	
		% DAPI positive cell	DNA laddering
*L. coromandelica* (LC)	Twig	79.5 ± 5.5	Positive
*C. tomentosa* (CT)	Twig	69.0 ± 6.5	Negative
*E. succirubrum* (ES)	Twig	77.0 ± 13.5	Negative
*D. castanea* (DC)	Leaves	72.5 ± 12.5	Negative
*D. castanea* (DC)	Twig	66.0 ± 11.5	Positive
*B. anceps* (BA)	Twig	68.5 ± 7.5	Negative
Melphalan	–	82.5 ± 8.0	Positive

### Apoptosis induction by plant extracts determined by DNA laddering assay

**Fig. 2:**
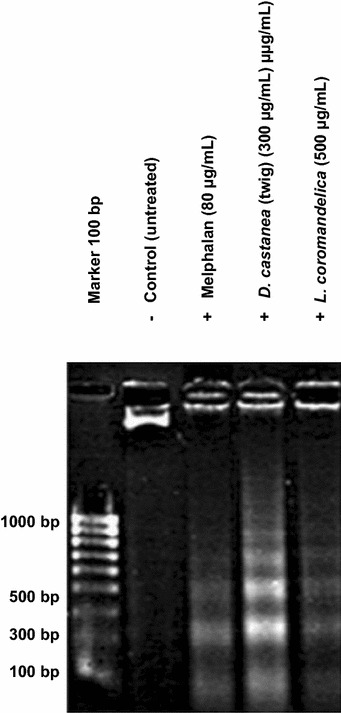
DNA fragmentation in treated HepG2 cells. DNA laddering visualized in HepG2 cells after 24 h exposed to 50 % ethanol–water crude extracts of *D. castanea* and *L. coromandelica*. Untreated cells and 80 μg/mL of melphalan were used as negative and positive controls, respectively

After treatment of HepG2 cell lines for 24 h with plant extracts, the DNA fragmentation of apoptotic cells was observed as DNA ladders using agarose gel electrophoresis. The results of DNA laddering showed that only LC and DC (twig) induced late-state apoptosis in HepG2 (Fig. 2); the other extracts did not induce late-stage apoptosis in HepG2 cells.

### Major components of DC (twig) and LC (twig) extracts using GC–MS analysis

The GC–MS chromatograms of the DC (twig) and LC (twig) extracts were examined. GC peaks with retention times of 29, 32, 39 and 52 min were found for the two crude extracts and were used for further chemical constituent identification using MS database analysis (Tables 4 and 5).

**Table 4:** Gas chromatography–mass spectrometry profile of 50 % ethanol–water crude extracts of *D. castanea* (twig)

Peak no.	Retention time (min)	% total area of prominent peak	Mass spectra	Compound
1	16.09	18.744	52, 63, 80, 97, 108, 117, 126	1,2,3-benzenetriol or pyrogallol
2	39.00	7.463	57, 74, 85, 98, 112, 123, 134, 154, 168, 182, 196, 213, 227, 239, 257, 270, 283, 299, 312, 330	2-palmitoylglycerol
3	52.73	5.824	55, 69, 81, 95, 109, 121, 135, 147, 161, 175, 189, 207, 218, 229, 243, 257, 272, 286, 297, 315, 326, 344, 357, 370, 393, 411, 426	(3β)-lup-20(29)-en-3-ol or lupeol

**Table 5:** Gas chromatography–mass spectrometry profile of 50 % ethanol–water crude extracts of *L. coromandelica* (twig)

Plants peak no.	RT (min)	% total area of prominent peak	Mass spectra	Compound
1	29.65	7.135	51, 60, 73, 83, 97, 107, 115, 129, 143, 157, 171, 185, 199, 213, 227, 239, 248, 256	Hexadecanoic acid or palmitic acid
2	30.05	5.013	55, 63, 73, 81, 88, 101, 108, 115, 122, 129, 136, 143, 150, 157, 167, 177, 185, 192, 199, 206, 213, 220, 227, 234, 241, 255, 263, 270, 284	Ethylhexadecanoate or ethyl palmitate
3	33.31	6.501	55, 65, 73, 83, 97, 111, 121, 129, 143, 155, 163, 171, 185, 199, 213, 222, 233, 241, 255, 264, 276, 284	Octadecanoic acid or stearic acid
4	37.77	8.621	55, 69, 81, 91, 105, 117, 129, 141, 155, 169, 183, 197, 211, 225, 239, 248, 247, 267, 276, 285, 300	Abieta-8,11,13-trien-18-oic acid or dehydroabietic acid
5	39.07	14.020	57, 74, 98, 112, 134, 154, 168, 182, 196, 213, 226, 239, 257, 270, 283, 330	2-palmitoylglycerol
6	42.14	6.166	57, 69, 84, 98, 112, 134, 154, 168, 185, 197, 210, 224, 241, 255, 267, 285, 298, 311, 327, 340, 358	Glyceryl monostearate

2-Palmitoylglycerol was the only major compound that co-existed in both the DC (twig) and LC (twig) crude extracts. Pyrogallol (1,2,3-trihydroxybenzene or 1,2,3-benzenetriol) and lupeol (3β-Lup-20(29)-en-3-ol) were the major compounds in DC (twig) crude extract. Hexadecanoic acid (abieta-8,11,13-trien-18-oic acid) and octadecenoic acid were the major compounds in the LC (twig) crude extract.

## Discussion

The five plants (LC, CT, ES, DC leaves, DC twig, and BA) used in this study are found in northeastern Thailand and were selected based on their ethnopharmacology. All the crude extracts showed significantly lower cytotoxicity than melphalan (*P* < 0.001, *P* < 0.001, *P* < 0.001, *P* = 0.002, *P* = 0.023 and *P* < 0.001, respectively). Based on their cytotoxicity and selectivity classification, the extracts could be categorized as follows: (a) potentially cytotoxic (IC_50_ < 10 μg/mL) with high selectivity (SI > 3); (b) moderately cytotoxic (IC_50_ < 100 μg/mL) with high selectivity (SI > 3); (c) moderately cytotoxic (IC_50_ < 100 μg/mL) with less selectivity (SI < 3); (d) cytotoxic to only normal cells with less selectivity; and (e) nontoxic [32]. Only the crude extract of DC (twig) could be classified as highly cytotoxic toward HepG2 cells and highly selective (SI = 4.5). The SI of this extract was not significantly different from melphalan (*P* = 0.959). DC (leaf) and other crude plant extracts had moderate cytotoxicity and less selectivity (SI < 3). The LC showed a cytotoxic effect against normal cells with low selectivity (SI < 3). To our knowledge, this study demonstrates for the first time the anticancer potential of LC extract against human HCC (HepG2) cancer cells. The extracts of CT, ES, and BA showed moderate cytotoxicity on HepG2 cells but with less selectivity.

Several chemotherapeutic compounds have been reported to induce apoptosis [36]. In apoptotic cells, specific DNA cleavage is evident on electrophoretic analysis as a characteristic ladder pattern resulting from the multiple DNA fragments of oligonucleosomal size (180–200 bp) [37]. The DNA ladder assay has a lower sensitivity for apoptosis detection [38] because ladder formation can only be clearly observed when the extent of oligonucleosomal cleavage is extensive. Although more than 50 % of the apoptotic cells were observed with the DAPI staining assay in all of the ethanolic plant extracts, only LC and DC were positive according to the DNA ladder assay.

It was necessary to standardize the two potential crude extracts for quality control experiments as 50 % ethanol–water crude extracts of DC (twig) and LC (twig) were used in the current study. We then determined which phytochemicals in the crude extracts were associated with the apoptosis effects using GC–MS analyses.

2-Palmitoylglycerol was the major compound common to the LC (twig) and DC (twig) crude extracts. 2-Palmitoylglycerol is involved in the regulation of endogenous cannabinoid receptor activity by increasing the biological activity of 2-arachidonylglycerol, and inhibition of adenyl cyclase 2-palmitoylglycerol significantly inhibits the inactivation of 2-arachidonylglycerol by neuronal and basophilic cells [39].

2-Arachidonylglycerol inhibits the production of TNF-alpha by mouse macrophages in vitro and the effect is enhanced in the presence of 2-palmitoylglycerol [40]. 2-Palmitoylglycerol is also a key component in modulating pain sensitivity as a result of its ability to interact with endocannabinoids [41]. In the present study, 2-palmitoylglycerol was the one compound found in both DC and LC extracts; however, the 2-palmitoylglycerol content was twice as low in the DC (twig) (7.5 %) than in the LC (twig) (14.0 %) and the DC extracts were more cytotoxic. It was not possible to specifically identify 2-palmitoylglycerol as the key compound; therefore, more detailed chemical identification of the plant extracts is required.

Pyrogallol was predominantly found (18.7 %) in the DC (twig) crude extract, which possessed high toxicity and selectivity against the HepG2 cells. Pyrogallol is a superoxide anion generator because it potently increases intracellular superoxide anion levels and decreases glutathione content in HeLa cells [42]. Research shows that pyrogallol-induced apoptosis resulting from the loss of mitochondrial membrane potential in calf pulmonary artery endothelial cells is accompanied by glutathione depletion [43]. In one study, the apoptosis induction of pyrogallol was indicated by a pyrogallol-type structure in a B-ring of catechins. DNA fragmentation activity was exhibited in a concentration-dependent manner in histiocytic lymphoma U937 cells after treatment with catechins containing a pyrogallol-type structure in a β-ring. Notwithstanding, catechins without a pyrogallol-type structure in any position did not show apoptosis-inducing activity [44]. A 3-*O*-gallate residue with *cis*-relationship to the β-ring enhanced the activity and the destruction of a pyrogallol-type structure by its methylation reduced this effect. By contrast, a 3-*O*-gallate residue in *trans*-relationship to the β-ring had little effect [44].

DC (twig) crude extract also contained lupeol as the third major compound (5.8 %). In a previous study, two hepatoma cell lines (SMMC7721 and HepG2) treated with lupeol decreased in a concentration-dependent manner (a) cell viability, (b) the induction of active caspase-3 and poly (ADP-ribose) polymerase cleavage, (c) cell accumulation in the S phase, and (d) apoptosis. Treatment with lupeol three times a week also resulted in chemosensitization of hepatoma cells and significantly inhibited tumor growth in nude mice implanted with SMMC7721 cells [45]. Lupeol chemosensitized hepatoma cells synergistically with a PI3-kinase inhibitor (S14161) in both in vitro and in vivo models [46]. In the present study, the lupeol content of the DC (twig) extract (5.8 %) was higher than that of LC (twig) extract (1.0 %). Pyrogallol was the major compound in the DC (twig) extract but not in the LC (twig) extract. These two major compounds might contribute to the high cytotoxicity and high SI of DC (twig) extract as well as the high toxicity with lesser selectivity of LC (twig) extract.

Hexadecanoic acid and octadecenoic acid were also identified as the major compounds in LC (twig) crude extract in this study. The crystal structure (at a resolution of 2.5 Å) and kinetics studies have shown that n-hexadecanoic acid can act as an anti-inflammatory compound by inhibiting phospholipase A_2_ in a competitive manner [47]. This fatty acid possibly contributes as the antihepatoma cell potential in the 50 % hydroethanolic herb extracts [13]. Pereira et al. [48] showed that the lipophilic extract from *Marthasterias glacialis* L. contained mainly palmitic acid and the sterol ergosta-7,22-dien-3-ol: these affected DNA synthesis, lipid droplets, and chromatin condensation, compatible with apoptosis in human breast cancer (MCF-7) and human neuroblastoma (SH-SY5Y) cell lines.

n-Hexadecanoic acid and 9,12-octadecadienoic acid (Z, Z) are fatty acids commonly found in the CO_2_ supercritical fluid extraction that mediates apoptosis in human hepatoma SMMC-7721 cells, involving a reactive oxygen species-mediated mitochondrial signaling pathway [49]. The mixture of an octadecenoic acid extract—comprising mainly oleic and linoleic acids—from *Euphorbia kansui* resulted in a concentration-dependent reduction in the number of human gastric (SGC-7901), HCC (BEL-7402), and leukemia (HL-60) cells and significantly inhibited cell proliferation, with induced apoptosis and G_0_/G_1_ phase cell cycle arrest [50]. The octadecenoic acids not only caused cell apoptosis/necrosis but also functional and structural damage to the tumor cell membrane and cell ultrastructures [50]. A fraction of the dichloromethane extract of *Protaetia brevitarsis* larva (composed of at least three free fatty acids: palmitic acid, (Z)-9-octadecenoic acid, and octadecenoic acid) expresses apoptosis-inducing activity as shown by DNA laddering and caspase-3 activation in colon 26 tumor cells [51].

## Conclusion

Ethanolic extracts from DC and LC twigs induced apoptosis in the HepG2 cell line. Pyrogallol and lupeol in DC (twig) might be responsible for the cytotoxicity toward the HepG2 cancer cells.

## Abbreviations

BA: *Bombax anceps* Pierre var. *anceps*
CT: *Catunaregam tomentosa* (Blume ex DC.) Tirveng.
ES: *Erythrophleum succirubrum* Gagnep.
LC: *Lannea coromandelica* (Houtt.) Merr.
DC: *Diospyros castanea* (Craib) Fletcher.
HCC: hepatocellular carcinoma
DMEM: Dulbecco’s modified Eagle’s medium
DMSO: dimethylsulfoxide
FBS: fetal bovine serum
GC-MS: gas chromatography/mass spectrometry
NR: neutral red
DAPI: 4′,6-diamidino-2-phenylindole
PBS: phosphate buffered saline
IC_50_: 50 % inhibitory concentration
SI: selectivity index
UV: ultraviolet
